# How *Salmonella* Survives the Macrophage’s Acid Attack

**DOI:** 10.1371/journal.pbio.1002117

**Published:** 2015-04-14

**Authors:** Lauren A. Richardson

**Affiliations:** Public Library of Science, San Francisco, California, United States of America

## Abstract

Instead of combating the acidification of the host cell vacuole, *Salmonella* acidifies its own cytoplasm in response to the low extracellular pH, helping it to secrete effector molecules.

Macrophages destroy bacteria by engulfing them in intracellular compartments, which they then acidify to kill or neutralize the bacteria. However, some pathogenic bacteria, such as *Salmonella enterica*, have evolved to exist and even grow while within these acidified compartments. Yet, how *Salmonella* responds to the acidic environment and how that environment affects the virulence of this pathogen are unclear.

In the paper presented here, published in *PLOS Biology*, Smarajit Chakraborty, Hideaki Mizusaki, and Linda Kenney demonstrate that, instead of combating the acidification of the *Salmonella*-containing vacuole (SCV), *Salmonella* acidifies its own cytoplasm in response to the extracellular low pH. The acidic cytoplasm then acts as a signal to stimulate the secretion of a particular class of *Salmonella* virulence proteins. These virulence proteins, or effectors, are released into the host cell, where they are able to perturb the immune response.

To investigate the effect of the acidic environment on *Salmonella*, Chakraborty and colleagues used a biosensor, called an I-switch, composed of a DNA strand with a different fluorophore ligated to each end. In the presence of excess hydrogen ions (i.e., acidic pH), the DNA strand bends, bringing the two fluorophores in closer proximity. The distance between the two fluorophores can be directly correlated to pH, and this can be measured by the change in emission wavelength. In this way, by inserting the I-switch into *Salmonella* cells, the authors were able to measure the pH of the cytoplasm simply by exciting the cells with one wavelength of light and then measuring the emission wavelength.

Their studies using the I-switch reveal that the *Salmonella* cytoplasm acidifies rapidly after entry into and acidification of the SCV. This acidification requires a pair of proteins, EnvZ and OmpR (Fig. 1). EnvZ is a sensor kinase that responds to intracellular osmotic stress. When activated, EnvZ phosphorylates OmpR, a DNA-binding protein that regulates the expression of numerous proteins. In *Salmonella* strains lacking either the EnvZ or the OmpR gene, the cytoplasmic pH remained near neutral, despite extracellular pH stress, indicating that the acidification of the cytoplasm is a regulated, not a passive, response. Blocking the acidification of the SCV by inhibiting the macrophage proton pump prevented the acidification of the *Salmonella* cytoplasm, demonstrating that the acidification is a direct response to the decreased extracellular pH.

**Fig 1:**
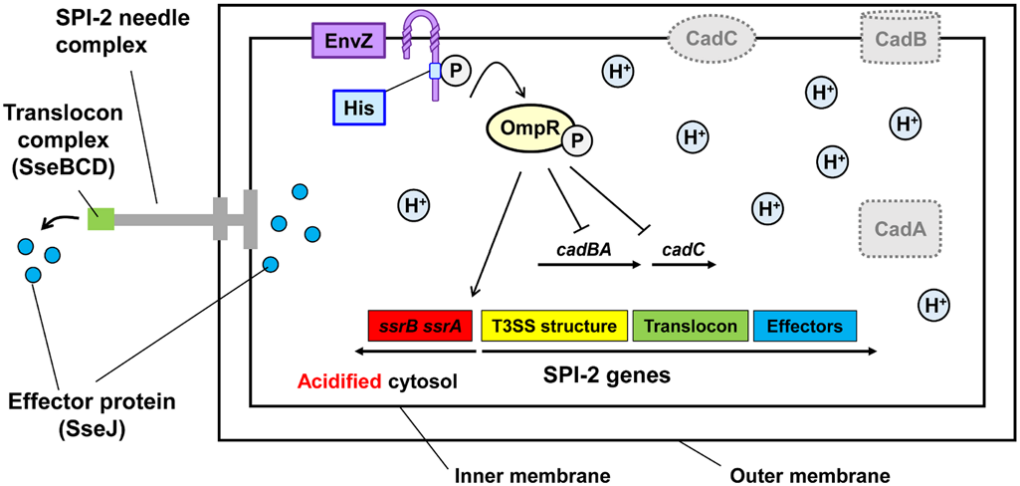
Inside the macrophage vacuole, EnvZ and OmpR are activated. OmpR represses cadC/AB, maintaining an acidic environment that facilitates expression of SPI-2. Effector proteins such as SseJ are secreted into the host, enabling *Salmonella* to replicate within the vacuole.

As mentioned above, OmpR regulates the expression of numerous genes, but the authors found that none of these known genes were responsible for mediating the acidification of the cytoplasm. Instead, they identified a new target, the cadC/BA operon (see Fig. 1). This operon encodes proteins that catalyze reactions that consume excess protons, and the expression of this operon is critical for maintaining intracellular pH homeostasis. The data indicate that OmpR acts as a repressor of this operon; thus, when OmpR is activated in the SCV, it stifles the normal response to low pH stress. Additionally, they find that OmpR increases the expression of genes encoding the *Salmonell*a ATP synthase, a large protein complex that pumps protons into the cell to create a proton gradient to produce ATP. By these two actions, repressing the acid stress response and increasing ATP synthase production, activation of OmpR mediates the acidification of the *Salmonella* cytoplasm following acidification of the SCV.

OmpR also regulates *Salmonella* pathogenicity island 2 (SPI-2), which encodes a nanomachine called the type III secretion system. This nanomachine is composed of a needle complex used to inject bacterial virulence proteins into the host cell. To determine if acidification of the *Salmonella* cytoplasm was required for the production of this nanomachine, the authors looked at the localization of one of the structural proteins, SseB, and one of the secreted virulence proteins, SseJ. They find that the needle complex is formed even if the cytoplasm remains neutral, but acidification is required for the complex to associate with the host vacuole membrane. Additionally, secretion of the virulence protein SseJ was blocked in the absence of acidification, demonstrating that the low pH of the cytoplasm is required for a successful intracellular *Salmonella* infection.

The findings of this paper contradict other previous reports that suggest that a neutralization step is required for secretion of the virulence proteins. The authors here show that *Salmonella* has adapted what was once an antibacterial response by the macrophage into a signal for when it is in the correct time and place to secret its virulence proteins and establish an infection.
